# Tumor Treating Fields: killing two birds with one stone

**DOI:** 10.1172/JCI159073

**Published:** 2022-04-15

**Authors:** Juyeun Lee, Matthew M. Grabowski, Justin D. Lathia

**Affiliations:** 1 Department of Cardiovascular & Metabolic Sciences, Lerner Research Institute and; 2 Rose Ella Burkhardt Brain Tumor and Neuro-Oncology Center, Cleveland Clinic, Cleveland, Ohio, USA.; 3 Case Comprehensive Cancer Center, Cleveland, Ohio, USA.

## Abstract

Given its aggressive natural history and immunosuppressive nature, glioblastoma (GBM) remains difficult to treat. Tumor Treating Fields (TTFields) are a promising treatment for GBM patients, yet the entirety of their antitumor action has not been fully elucidated. In a recent issue of the *JCI*, Chen et al. explored the effect of TTFields in reinvigorating immune responses. By elegant step-by-step approaches, the authors demonstrated that TTFields promote the production of immune-stimulating proinflammatory and interferon type 1 cytokines in tumor cells in a cGAS/STING- and AIM2 inflammasome–dependent mechanism, thereby activating the immune system. The findings show that TTFields not only directly inhibit tumor cell growth, as previously reported, but enhance antitumor immunity, suggesting TTFields can be used as an immune-modulating approach in GBM.

## The immunosuppressive nature of glioblastoma

Glioblastoma (GBM) is the most aggressive and most common primary adult brain cancer, accounting for over 80% of primary malignant brain and other CNS tumors (1). These patients have a dismal prognosis, with a median survival of approximately 20 months and one- and five-year survival rates of only 35.0% and 4.7%, respectively (2). Therapies that improve survival in GBM are scarce, with the current standard of care consisting of surgery, radiotherapy, and chemotherapy with temozolomide (TMZ) (3). The disease produces many challenges that negatively impact patient well being and lowers treatment efficacy. Interfering attributes include high cellular proliferation, infiltration, inflammation, resistance to apoptosis, angiogenesis, and widespread genomic alterations, and are confounded by profound immunosuppressive effects both locally within the tumor microenvironment (TME) and systemically in the body (4–6). The complex immune interplay involves dysfunctional antitumor immune cells, such as T and NK cells; expansion of antiinflammatory immune cells, such as regulatory T cells, myeloid-derived suppressor cells (MDSCs), and protumorigenic, polarized, glioma-associated macrophages and microglia; upregulation of tumor- and hypoxia-associated immunosuppressive cell surface factors and cytokines; and iatrogenic sequelae of immunosuppressive treatments (1, 4). For any treatment to show efficacy in halting tumor progression, it will likely require mechanisms that combat the disease at the cellular, TME, and systemic levels simultaneously.

## TTFields and GBM

First available in 2011 for the treatment of recurrent GBM, Tumor Treating Fields (TTFields) have shown a modest but statistically significant increase in progression-free and overall survival (6.7 vs. 4.0 months, and 20.9 vs. 16.0 months, respectively, for TTFields with TMZ versus TMZ alone) in a randomized, multicenter trial (2). TTFields are generated by a portable, battery-powered device that is worn by the patient for at least 18 hours a day, and requires patients to maintain a shaved head. The rationale for its efficacy has been explained by the dividing-cell destruction and arrest of proliferation when applying a properly oriented, very low intensity, intermediate frequency, alternating electric field to tumor cells (7, 8). At a subcellular level, TTFields disrupt the polymerization-depolymerization process of charged tubulin subunits and thereby interfere with formation of the mitotic spindle during mitosis (7, 8). Beyond this initial tumor cell cycle–dependent effect, recent publications describe evidence of positive immune activation within the TME and systemically in both animal models and human patients (9–11); however, a mechanistic rationale for these observations is unclear.

## TTFields activate cGAS/STING and AIM2 inflammasome pathways

**Figure 1:**
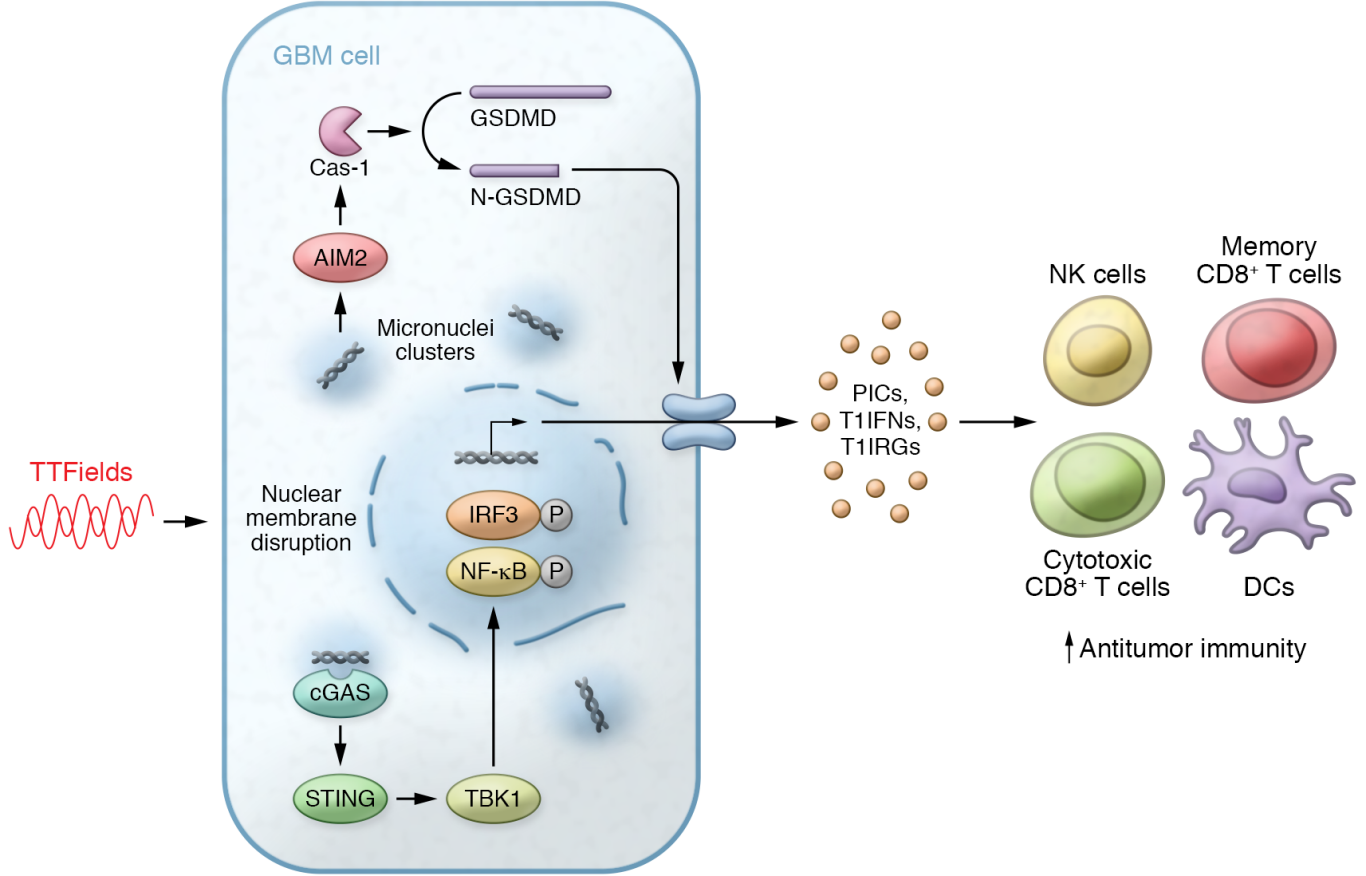
Molecular and cellular mechanisms of TTFields effects on GBM. TTFields induce production of PICs and T1IFNs in cGAS- and AIM2-mediated ways, thereby enhancing anti-GBM immune responses. TTFields, tumor-treating fields; GBM, glioblastoma; TBK1, TANK-binding kinase 1; IRF3, interferon regulatory factor 3; Cas-1, caspase-1; GSDMD, gasdermin D; PICs, proinflammatory cytokines; T1IFNs, type 1 interferons; T1IRGs, type 1 interferon–responsive genes.

In this issue of the *JCI*, Chen et al. uncovered tumor cell responses upon TTFields administration. The authors used murine GBM models and samples from patients to describe TTFields-imposed molecular and immune effects (Figure 1 and ref. 12). Based on previous studies showing the possible link between TTFields and immune activation (9, 11), the authors hypothesized that cytosolic micronuclei induced by TTFields may activate inflammasome signaling pathways. Indeed, TTFields resulted in increased cytosolic micronuclei clusters in the tumor cells accompanied with upregulated transcription of proinflammatory cytokines (PICs), type 1 interferons (T1IFNs), and T1IFN-responsive genes (T1IRGs). Mechanistically, the upregulated cytokine production was abrogated when mice lacked STING via knockdown. Conversely, overexpression of STING rescued cytokine production, indicating that the cGAS/STING pathway plays a key role in TTFields’ downstream effects in tumor cells (Figure 1). TTFields also induced activation of AIM2 inflammasomes, which subsequently activated caspase-1. Notably, caspase-1 cleaves gasdermin D (GSDMD) and the cleaved N-terminal domain forms transmembrane pores that are required for the release of inflammatory cytokines (13). Knocking down AIM2 prevented cleavage of GSDMD upon TTFields administration, further confirming TTFields-mediated activation of AIM2 inflammasomes (Figure 1). Additionally, TTFields enhanced cell death measured by LDH release. The authors claimed that the cell death was due to TTFields-mediated membrane damage and less likely linked to late apoptosis, which can be induced by TMZ treatment. Clinical trials that tested TTFields therapy in GBM together with TMZ administration showed promising results, with prolonged progression-free survival and overall survival (2). Thus, understanding the precise mechanisms of cell death triggered by TTFields could provide better insights to improve combination therapies.

## TTFields enhance antitumor immune responses

Chen and colleagues demonstrated that TTFields enhanced immune responses against GBM using in vitro and in vivo approaches (12). The in vitro investigation showed that TTFields-treated tumor cells produced signaling molecules, presumably PICs and T1IFNs, which can induce activation of immune cells in a STING- and/or AIM2-dependent manner. Subsequent in vivo experimental models showed that the enhanced survival noted for animals implanted with TTFields-treated wild-type (WT) tumor cells was abrogated when mice were challenged with TTFields-treated STING/AIM2-double-knockdown tumor cells. T1IFN supports T cell priming and delivers costimulatory signals that determine the fate of T cells to become memory populations (14). As such, TTFields-treated WT tumor cells induced robust memory immune responses and protected animals from rechallenge, with upregulated responses in DCs and T cells as well as increased T1IFN signaling in the TME and the periphery. Notably, the authors treated GBM tumor cells with TTFields before implanting them into mouse models to separate the tumor-intrinsic effect from the direct effects of TTFields on stromal cells. The results from mouse models aligned well with human data, yet further investigation of the effect of TTFields on the TME may be necessary to fully understand the mechanism of TTFields underlying the immune activation. Chen and colleagues further corroborated their findings by examining the GBM transcriptome of peripheral blood mononuclear cells from patients with GBM before and after TTFields. Single-cell RNA-sequencing analysis revealed an increase in specific immune cell populations, including plasmacytoid DCs, monocytes, and NK cells, which respond to PIC and T1IFN signals (Figure 1). A decrease in an exhausted CD8^+^ T cell population was found, while memory T cell formation increased, supporting the possibility of an enhanced memory response upon TTFields administration, as observed in mouse models (Figure 1). The authors also analyzed T cell receptor (TCR) diversity from deep RNA sequencing on T cells isolated from patients’ blood. Most of the patients (9 of 12) showed clonal expansion of T cells after TTFields therapy, indicating stronger tumor-specific immune responses, as TCR diversity negatively correlates with expression levels of cytotoxic cytokines, regulatory genes, and immune checkpoint inhibitors.

## Conclusions and future directions

The current study by Chen et al. demonstrated the precise molecular mechanisms by which TTFields regulates immune responses and possibly enhances antitumor immunity (12). Several questions arise from these initial findings. Combining TTFields and TMZ treatment has been tested in clinical trials and shown to improve prognosis of patients with GBM (2). Previously, TMZ has been reported to have a lymphotoxic effect (15). Another study showed that TMZ treatment enhanced tumor-specific immune responses (16). Given the opposite effects of TMZ on the immune system, the immune-enhancing effect of TTFields needs to be evaluated in combination with TMZ. Moreover, as these data suggest that TTFields can enhance immune activation, follow-up studies combining TTFields with approaches to reduce TME immune suppression, such as anti-MDSC or anti–tumor-associated macrophage (anti-TAM) therapies, should be considered. It is also of note that Chen et al. focused exclusively on peripheral immune responses. Future studies need to address whether TTFields reinvigorate the largely suppressed antitumor immunity in the TME. In addition, TTFields were reported to disrupt the integrity of the blood brain barrier (17), suggesting changes in immune cell infiltration into the brain, and this further suggests a more detailed interrogation of the TME with TTFields alone, and in combination with several other therapies listed above. Taken together, Chen et al. inspire further investigation and provide the foundation for harnessing antitumor immunity in the treatment of GBM and other solid tumors.

## Version 1 04/15/2022

Electronic publication
